# ALG3-CDG: a patient with novel variants and review of the genetic and ophthalmic findings

**DOI:** 10.1186/s12886-021-02013-2

**Published:** 2021-06-05

**Authors:** Martina Farolfi, Anna Cechova, Nina Ondruskova, Jana Zidkova, Bohdan Kousal, Hana Hansikova, Tomas Honzik, Petra Liskova

**Affiliations:** 1 Department of Paediatrics and Inherited Metabolic Disorders, First Faculty of Medicine, Charles University and General University Hospital in Prague, Ke Karlovu 2, 128 08 Prague, Czech Republic; 2 Centre of Molecular Biology and Genetics, University Hospital Brno and Masaryk University, Brno, Czech Republic; 3 Department of Ophthalmology, First Faculty of Medicine, Charles University and General University Hospital in Prague, Prague, Czech Republic

**Keywords:** N-linked glycosylation, Congenital disorder of glycosylation, ALG3-CDG, Optic nerve hypoplasia, Arthrogryposis, Transferrin isoelectric focusing, Novel mutation

## Abstract

**Background:**

ALG3-CDG is a rare autosomal recessive disease. It is characterized by deficiency of alpha-1,3-mannosyltransferase caused by pathogenic variants in the *ALG3* gene. Patients manifest with severe neurologic, cardiac, musculoskeletal and ophthalmic phenotype in combination with dysmorphic features, and almost half of them die before or during the neonatal period.

**Case presentation:**

A 23 months-old girl presented with severe developmental delay, epilepsy, cortical atrophy, cerebellar vermis hypoplasia and ocular impairment. Facial dysmorphism, clubfeet and multiple joint contractures were observed already at birth. Transferrin isoelectric focusing revealed a type 1 pattern. Funduscopy showed hypopigmentation and optic disc pallor. Profound retinal ganglion cell loss and inner retinal layer thinning was documented on spectral-domain optical coherence tomography imaging. The presence of optic nerve hypoplasia was also supported by magnetic resonance imaging. A gene panel based next-generation sequencing and subsequent Sanger sequencing identified compound heterozygosity for two novel variants c.116del p.(Pro39Argfs*40) and c.1060 C > T p.(Arg354Cys) in *ALG3*.

**Conclusions:**

Our study expands the spectrum of pathogenic variants identified in *ALG3*. Thirty-three variants in 43 subjects with ALG3-CDG have been reported. Literature review shows that visual impairment in ALG3-CDG is most commonly linked to optic nerve hypoplasia.

## Background

Congenital disorders of glycosylation (CDG) are a rapidly expanding group of genetic multisystem diseases characterized by hypoglycosylation of glycoproteins and glycolipids. Based on the type of glycosidic bond, N- and O-glycosylation disorders are distinguished as two main subclasses of CDG [1].

ALG3-CDG (MIM #601,110) is a rare autosomal recessive disorder of protein N-glycosylation caused by the deficiency of alpha-1,3-mannosyltransferase, which adds one mannose residue in an alpha-1,3 linkage to Man5GlcNAc2-PP-Dol [2].

ALG3-CDG patients show various combinations of cardiac defects (obstructive cardiomyopathy, dilatation of aorta), lung hypoplasia, hepatomegaly, neurological impairment (cerebral and cerebellar atrophy, agenesis of corpus callosum, epilepsy) with developmental delay, musculoskeletal involvement (ulnar deviation, joint contractures, skeletal dysplasia, scoliosis), urogenital abnormalities (nephrocalcinosis, renal cysts, hydronephrosis), microcephaly, visual impairment and dysmorphic features (down slanting palpebral fissures, hypertelorism, high nasal bridge, anteverted nares, large and thick low-set ears with abnormal pinnae, micrognathia, thin lips, inverted nipples). Forty-two cases of ALG3-CDG have been reported to date [2–18].

In this study, we characterize in detail the first Czech patient with ALG3-CDG and provide a review of the literature on the identified disease-causing variants and reported ocular findings.

## Case presentation

The study was approved by the Ethics committee of the General University Hospital in Prague and adhered to the tenets of the Helsinki Declaration.

The patient was born at 34 6/7 weeks of gestation, with a birth weight of 2.190 kg (34th percentile, -0.41 SD), length of 45 cm (46th percentile, -0.1 SD) and head circumference of 30 cm (16th percentile, -1 SD), following a pregnancy with polyhydramnion and discrete pericardial effusion. She is the first child of non-consanguineous Czech parents. Her early postnatal adaptation was uneventful (Apgar score 8-9-9), but already at birth multiple joint contractures and craniofacial dysmorphic features were noticed. The otoacoustic emissions were absent. Abdominal ultrasound documented multiple renal cysts.

**Fig. 1:**
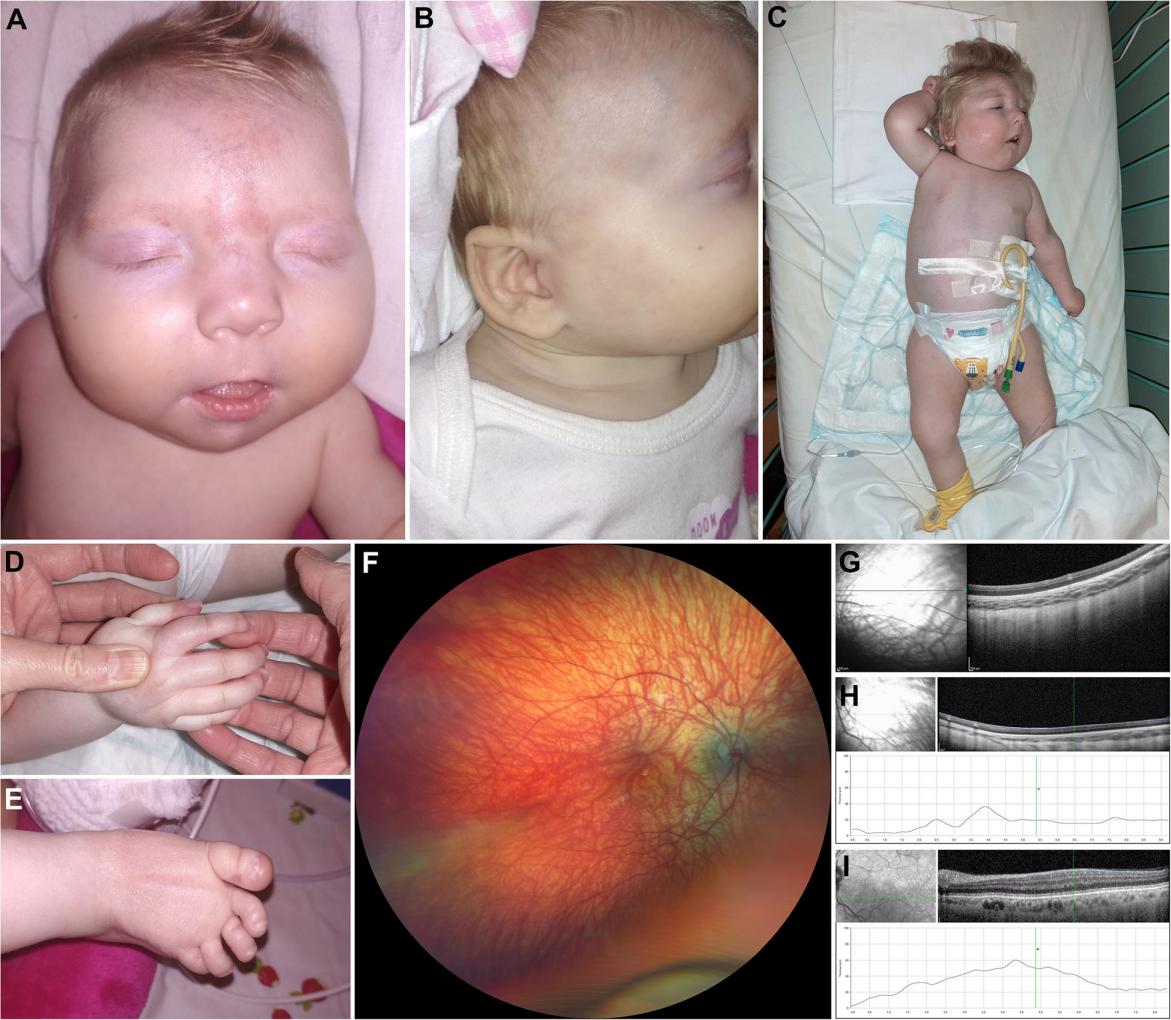
Clinical findings in the Czech case with ALG3-CDG. Photograph of the girl at the age of 4 months (**A**, **B**) and at the age of 23 months (**C**-**E**). Note microcephaly, port-wine stain on the forehead, hypertelorism, wide and flattened nasal bridge, long and smooth philtrum, thin upper lip (**A**), dysplastic and low set ears with malformed pinnae (**B**), down-slanting palpebral fissures, mild ptosis and anteverted nares, contracted wrists and long fingers, semiflexed knees, gastrostomy (**C**). Detailed photographs of the hand and leg showing flexed joints, long fingers with ulnar deviation, adducted thumbs (**D**), rigid clubfoot, medial protrusion of the navicular bone, lateral deviation of high arch and toes (**E**). Wide-field fundus photograph of the right eye taken at the age of 23 months showing optic nerve hypoplasia and hypopigmentation (**F**). SD-OCT horizontal perimacular scan of the left eye (**G**), note inner retinal layer thinning together with preservation of the external limiting membrane, ellipsoid zone and interdigitation zone. Analysis of the ganglion cell layer using automatic segmentation documents profound loss (**H**) when compared to a scan obtained from a similarly aged healthy female child (**I**). The quality of retinal imaging was limited by compliance, images of the left eye could not be obtained

At 3 months of age, the girl was admitted to the hospital with recurrent apnoeic episodes, congenital laryngeal stridor and micro-aspirations. Oral feeding was found to be risky and gastrostomy was placed. On clinical examination she presented with central hypotonia, plagiocephaly, abnormal subcutaneous fat distribution around the neck, port-wine stain on the forehead and craniofacial dysmorphia (Fig. 1A, 1B). Length (55 cm, 12th percentile) and weight (4.66 kg, 39th percentile) were normal but there was severe progressive microcephaly (head circumference 34.5 cm, <1st percentile, -3.49 SD). Laboratory investigations showed antithrombin (35 %, controls 80–140 %) and factor XI deficiency (42 %, controls 55–135 %).

Neurological examination showed spastic tetraplegia, hyporeflexia and developmental delay. Although clinical seizures were not noticed, electroencephalography (EEG) was severely abnormal showing high amplitude slow spike-wave complexes (SSWC) and slow background activity. Anticonvulsant therapy was therefore initiated. The brain magnetic resonance imagining showed cerebral and cerebellar atrophy, cavum septum pellucidum, enlarged 4th ventricle and cisterna magna, delayed myelinization, and optic nerve hypoplasia.

Ophthalmic examination showed hypopigmentation of the fundus and pale optic discs. Visual and brain stem auditory evoked potentials (VEP, BAEP) were both pathological with prolonged latencies of wave N70 and waves III, V, respectively. Cardiological examination was normal apart from insignificant patent arterial duct and patent foramen ovale.

At 12 months of age, epilepsy was not compensated despite combined therapy with levetiracetam and phenobarbital. Add-on therapy with topamirate was started. At that time, although the development was severely delayed, the patient began to fix objects and to react to sounds and voices of family members, gurgle and lift up her head.

Despite gastrostomy feeding, all her anthropometric parameters severely declined with length 66 cm (< 1st percentile, -2.35 SD), weight 7.38 kg (6th percentile, -1.96 SD) and head circumference 38 cm (< 1st percentile, -5.17 SD). EEG at 14 months showed similar pattern to the earlier recordings with slow background activity and multiple SSWC.

At the last follow-up, the girl was 23 months-old, severe developmental delay was apparent, seizures were ameliorated on triple anticonvulsant medication. Her recurrent micro-aspirations were life threatening. She showed further deceleration of the anthropometric data: length 77 cm (< 1st percentile, -2.69 SD), weight 10.2 kg (6th percentile, -1.96 SD) and head circumference 40 cm (< 1st percentile, -5.53 SD). On physical examination, flexed joints, long fingers with ulnar deviation, adducted thumbs and laterally deviated toes were observed (Fig. 1 C-1E).

We performed esophagogastroduodenoscopy that revealed esophagitis and duodenum ulcer. Repeated abdominal ultrasonography documented multiple cysts in both kidneys which were of stable size since birth, other abdominal organs were without abnormalities.

Ocular examination including spectral-domain optical coherence tomography (SD-OCT) (Spectralis, Heidelberg Engineering GmbH, Heidelberg, Germany) revealed profound loss of retinal ganglion cells and inner retinal layer thinning with preservation of the external limiting membrane, ellipsoid zone and interdigitation zone (Fig. 1 F-1I). Fundus appeared hypopigmented and optic discs were pale as documented using ultra-wide field camera (Clarus 700, Carl Zeiss Meditec AG, Jena, Germany). Visual activity could not be measured.

Transferrin isoelectric focusing was performed as described [19] and showed a type 1 pattern.

DNA from the proband and her parents was extracted from peripheral leucocytes. Targeted sequencing of 260 genes related to neuromuscular diseases and arthrogryposis was performed by hybrid capture enrichment (Roche NimbleGen, Pleasonton, CA, USA) and massive parallel sequencing (NextSeq, Illumina, San Diego, CA, USA) as described [20]. Sequencing data were analysed with Sequence Pilot software (JSI medical systems, Ettenheim, Germany). Conventional Sanger sequencing was used to verify the presence of presumed causal variants and for targeted screening in first-degree relatives. The effect of one missense mutation was predicted using five computational tools: Sorting Intolerant from Tolerant (SIFT) [21], Polyphen-2 [22], Mutation Taster2 [23], M-CAP [24] and CADD [25]. The pathogenicity of the detected variants was evaluated according to the American College of Medical Genetics and Genomics (ACMG) recommendations [26].

Two *ALG3* variants (NM_005787.6) c.116del p.(Pro39Argfs*40) and c.1060 C > T p.(Arg354Cys) were identified, neither of them has been reported. Segregation analysis confirmed their trans position, the c.116del was inherited from the mother while the c.1060 C > T from the father. All five prediction tools used evaluated the missense variant as deleterious or likely deleterious. Based on the available evidence both variants were classified as pathogenic according to the ACMG criteria.

## Discussion

In this study we describe clinical, biochemical, and molecular genetic findings of an unreported ALG3-CDG patient. We also review ocular and molecular genetic findings in all 43 individuals with ALG3-CDG identified to date.

Our case adds to the previously published phenotype data several novel aspects. In earlier reports pale or small optic disc was observed in 12 patients suggesting optic nerve atrophy or hypoplasia [2, 5, 6, 8, 9, 11, 14, 18]. Our case report is however the first clearly documenting optic nerve and retinal layers pathology by ocular imaging methods.

### Clinical course

Out of the 43 reported individuals 17 had a lethal phenotype with prenatal manifestation, medical termination of pregnancy and/or early neonatal death. Three further patients died of respiratory failure during pneumonia in childhood (aged 1.8; 3.5 and 6 years) [11, 12, 14] and one of multiorgan failure (aged 1 year) [18]. Neurological symptoms have recently been summarized in 26 patients, and a ketogenic diet was suggested as a therapeutic option for intracrable epilepsy [16]. Dysmorphic and musculoskeletal features have been also recently reviewed in 19 patients [15].

The longest living patient is 38 years-old male suffering from moderate intellectual disability, intractable seizures, feeding difficulties, facial dysmorphism, hearing loss, cardiac abnormalities, osteopenia/osteoporosis, nocturnal apnoea, scoliosis, hypothyroidism [18].

### Ocular phenotype

Presumably because of poor compliance due to young age at examination and developmental delay available ophthalmic data in ALG3-CDG are limited (Table 1). Most often poor eye fixation, strabismus and/or nystagmus have been noted. However, the likely underlying cause was only reported in few patients and comprised optic nerve atrophy/hypoplasia observed in 11 patients including the current study confirming deficiency of retinal ganglion cells by SD-OCT, and signs of chorioretinal dystrophy in three patients, of these one had also optic atrophy [11]. Electroretinography was reported in three patients; decreased amplitudes of both rod and cone responses were observed [4, 7].

**Table 1:** Summary of ocular and vision-related findings in patients with ALG3-CDG

Reference	Age at evaluation	Clinical findings
Korner [2]	5 y	Coloboma of the iris, optic nerve atrophy
Denecke [4]	6 y	Rotational and horizontal nystagmus; unable to fixate, recognize objects, pupillary light reflex present, reduced amplitudes on ERG (at 6 m)
	Pregnancy (19 w)	NR
Schollen [5]	4 y	Blindness, optic nerve atrophy
Sun [6]	Shortly after birth (36 w)	Bilateral optic nerve atrophy; abnormal pupillary light reflex
Kranz [7]	9 y	Cortical blindness, strabismus, decreased amplitudes of both rods and cones on ERG
	7 y	Strabismus, deposition of abnormal metabolic products in conjunctiva possibly indicating retinal involvement, decreased amplitudes of both rods and cones on ERG
Rimella-Le-Huu [8]	15 m	Poor visual contact; latent nystagmus; hypopigmentation of the retina, optic nerve atrophy
Riess [9]	15 y	Cortical visual impairment, divergent strabismus, myopia, mild optic nerve atrophy, normal retina, no cataracts, no nystagmus.
	21 y	Cortical visual impairment, strabismus, horizontal nystagmus, mild optic nerve atrophy, normal retina, no cataracts
Lepais [10]	MTP (25 w)	Ocular proptosis, corneal opacities
	Shortly after birth (36 w)	Bilateral congenital cataract
Fiumara [11]	2 y	Initial signs of chorioretinal dystrophy, i.e., optic nerve atrophy
Barba [12]	5 y	Poor eye contact
	6 y	Poor eye contact
Alsubhi [13]	Neonatal	NR
	Neonatal	NR
	Neonatal	NR
	Neonatal	NR
	Neonatal	NR
	Neonatal	NR
	Neonatal	NR
Himmelreich [14]	2 m	Descending eyelid axes, not evident fixation
	2 m	Descending eyelid axes
	3 y	NR
	16 m	Horizontal nystagmus and difficult fixation (noted at 3.5 m), on MRI (at 11 m) hypogenesis of the anterior optic pathways (optic nerve and chiasma), small papillae (at 11 m), able to see smaller objects
Bian [15]	MTP (28 w)	Eyelid ptosis
	MTP (22 w)	NR
Paketci [16]	4.5 m	Poor eye contact, deviations in the eyes
	2 m	Poor eye contact
Ferrer [17]	Fetal demise (24 4/7 w)	NR
	Stillbirth (30 1/7 w)	NR
Alsharhan [18]	17 y	Strabismus, myopia, thick eyebrows and eyelashes
	5 y	Optic nerve atrophy, epicanthal folds, long eyelashes, telecanthus
	2 y	Epicanthal folds, cortical blindness, small optic nerves chiasm and tracks
	7 y	Strabismus, myopia, impaired visual awareness
	Stillbirth	Down-slanting palpebral fissures
	1 y	Optic nerve atrophy
	30 y	Strabismus, epicanthal folds
	38 y	Down-slanting palpebral fissures
	36 y	Not detected
	Neonatal	NR
Current study	23 m	Poor fixation, down-slanting palpebral fissures, hypopigmented fundus, retinal ganglion cell loss, optic nerve hypoplasia

*ERG* electroretinography, *MRI* magnetic resonance imaging, *MTP* medical termination of pregnancy, *NR* not reported, *m* months, *w* weeks, *y* years

Ocular features reported including negative findings as provided in the original reports are shown. In most patients detailed ophthalmic examination has not been reported thus the presence of other phenotypes cannot be excluded

Ophthalmic examination in patients reported by Alsharhan [19] was assumed to be done at the same time as Nijmegen Pediatric CDG Rating Scale evaluation

Less common ocular features of ALG3-CDG were congenital cataract found in one individual [10] and iris coloboma present in another patient [2]. One fetus examined after pregnancy termination at week 25 was noted to have proptosis and corneal opacities [10]. Another fetus terminated in week 28 had eyelid ptosis [15].

### Disease-causing variants

Sequence variants previously reported as causing ALG3-CDG (reference sequence NM 005787.6) were searched in the literature. The population frequency of the variants was retrieved from the Genome Aggregation Database v2.1.1 from 125,748 human exomes and 15,708 genomes [27]. In total 33 pathogenic/likely pathogenic variants in *ALG3* have been found in 43 individuals with ALG3-CDG from 29 families (Table 2). Most subjects carried a unique disease-causing variant(s), only three mutations have been identified recurrently. Homozygous variants were mainly linked to consanguinity when this information was available [2–18].

**Table 2:** Summary of reported *ALG3* pathogenic/likely pathogenic sequence variants

Reference	DNA change	Protein change	Zygosity	gnomAD allele frequency	No of affected subjects	Origin and/or ethnicity
Korner [2]	c.353G>A	p.(Gly118Asp)	HOM	0	1	German
Denecke [3], Denecke [4]	c.165 C>T^a^	p.Val54Thrfs*13	HOM	0	2	Italian
Schollen [5]	c.796 C>T	p.(Arg266Cys)	HOM	1/248,496	1	White
Sun [6]	c.512G>A	p.(Arg171Gln)	HOM	9/279,202	1	Dominican Republic
Kranz [7]	c.211T>C c.470T>A	p.(Trp71Arg) p.(Met157Lys)	HET HET	0 0	2	White
Rimella-Le-Huu [8]	c.116 C>T c.512G>A	p.(Pro39Leu) p.(Arg171Gln)	HET HET	0 9/279,202	1	Swiss/Italian
Riess [9]	c.206T>C c.626T>C	p.(Ile69Thr) p.(Met209Thr)	HET HET	0 0	2	Vietnamese
Lepais [10]	c.286G>A	p.(Gly96Arg)	HOM	5/248,964	2	Turkish
Fiumara [11]	c.564_566del c.[1125G>A; c.1127del]	p.(Leu190del) p.[(Met375Ile; Pro376Leufs*92)]	HET HET	0 2/248,976;0	1	NR
Barba [12]	c.1 A>G	p.(Met1?)	HOM	1/189,664	1	NR
	c.165C>T^a^ c.1061G>A	p.Val54Thrfs*13 p.(Arg354His)	HET HET	0 5/248,666	1	NR
Alsubhi [13]	c.512G>A	p.(Arg171Gln)	HOM	9/279,202	7	Saudi Arabian
Himmelreich [14]	c.165 C>T^a^	p.Val54Thrfs*13	HOM	0	1	Turkish
	c.1263G>A	p.(Trp421*)	HOM	0	1	Iraqi
	c.165 C>T^a^ c.350G>C	p.Val54Thrfs*13 p.(Arg117Pro)	HET HET	0 1/248,826	1	Albanian
	c.296+4G>A^b^ c.1037 A>G	p.Tyr66Cysfs*43 p.(Asn346Ser)	HET HET	0 0	1	French
Bian [15]	c.512G>T c.511 C>T	p.(Arg171Leu) p.(Arg171Trp)	HET HET	1/247,844 4/247,478	2	Chinese
Paketci [16]	c.165 C>T^a^	p.Val54Thrfs*13	HOM	0	2	NR
Ferrer [17]	c.1188G>A	p.(Trp396*)	HOM	0	2^c^	Pakistani
Alsharhan [18]	c.656T>C c.749T>A	p.(Leu219Pro) p.(Leu250Gln)	HET HET	0 0	1	White
	c.796 C>T	p.(Arg266Cys)	HOM	1/248,496	1	Ecuador
	c.796 C>T	p.(Arg266Cys)	HOM	1/248,496	1	Ecuador
	c.991 C>T c.914 C>A	p.(Gln331*) p.(Ala305Asp)	HET HET	0 2/247,836	1	African American
	c.512G>T	p.(Arg171Leu)	HOM	9/279,202	1	Arabic
	c.611 C>T c.1154G>C	p.(Ala204Val) p.(Arg385Thr)	HET HET	0 0	1	African American
	c.72G>A c.521 A>G	p.(Trp24*) p.(Asn174Ser)	HET HET	0 0	1	White
	c.395 A>G c.752T>C	p.(Tyr132Cys) p.(Leu251Pro)	HET HET	1/249,172 0	2	White
	c.410_411insTGTCTTCTTGCT	p.(Leu137_Leu138insValPheLeuLeu)	HOM	0	1	Saudi Arabian
Current study	c.116del c.1060 C>T^d^	p.(Pro39Argfs*40) p.(Arg354Cys)	HET HET	0 6/248,692	1	Czech

*HET* heterozygous, *HOM* homozygous, *N* no, *NR* not reported, *Y* yes

^a^Predicted at protein level to be silent, i.e. p.(=), the variant was however shown at cDNA level to lead to deletion of 37 bp (r.160_196del) with aberrant splicing and introduction of premature termination codon

^b^At cDNA level leading to exon 2 deletion (r.197_296del)

^c^Both also carried a homozygous variant of uncertain significance c.944C>G p.(Ser315Cys) in *COG5*; OMIM # 6136122

^d^The variant is listed in the Euroglycanet network database (http://www.euroglycanet.org)

Each row represents a single family. Information on ethnicity and origin is as complete as it was possible to extract from published studies. Mutation description follows Human Genome Variation Society guidelines and NM_005787.6 was taken as the reference sequence. Allele frequency was mined from gnomAD v2.1.1

## Conclusions

CDG pose a diagnostic challenge due to their high phenotypic variability. Combination of severe neurologic and visual impairment with dysmorphia, arthrogryposis and other congenital malformations should raise suspicion of a CDG syndrome.

## Data Availability

All data generated or analysed during this study are included in this published article.

## Abbreviations

ACMG: American College of Medical Genetics and Genomics
ALG3: Asparagine-linked glycosylation 3
BAEP: Brain stem auditory evoked potentials
CDG: Congenital disorders of glycosylation
gnomAD: Genome aggregation database
EEG: Electroencephalography
MIM: Mendelian Inheritance in Man
SD: Standard deviation
SD-OCT: Spectral-domain optical coherence tomography
SSWC: Slow spike-wave complexes
VEP: Visual evoked potentials
